# Associations of recurrent miscarriages with chromosomal abnormalities, thrombophilia allelic polymorphisms and/or consanguinity in Saudi Arabia

**DOI:** 10.1186/s12881-016-0331-1

**Published:** 2016-10-10

**Authors:** Rola F. Turki, Mourad Assidi, Huda A. Banni, Hanan A. Zahed, Sajjad Karim, Hans-Juergen Schulten, Muhammad Abu-Elmagd, Abdulrahim A. Rouzi, Osama Bajouh, Hassan S. Jamal, Mohammed H. Al-Qahtani, Adel M. Abuzenadah

**Affiliations:** 1 Obstetrics and Gynecology Department, King Abdulaziz University Hospital, Jeddah, Saudi Arabia; 2 Center of Innovation in Personalized Medicine, King AbdulAziz University, P.O. Box: 80216, Jeddah, 21589 Kingdom of Saudi Arabia; 3 Center of Excellence in Genomic Medicine Research, King Abdulaziz University, Jeddah, Saudi Arabia

**Keywords:** Recurrent pregnancy loss, Chromosomal aberrations, Thrombophilia, Consanguinity, Cytogenetic analysis

## Abstract

**Background:**

Recurrent pregnancy loss (RPL) or recurrent spontaneous abortion is an obstetric complication that affects couples at reproductive age. Previous reports documented a clear relationship between parents with chromosomal abnormalities and both recurrent miscarriages and infertility. However, limited data is available from the Arabian Peninsula which is known by higher rates of consanguineous marriages. The main goal of this study was to determine the prevalence of chromosomal abnormalities and thrombophilic polymorphisms, and to correlate them with RPL and consanguinity in Saudi Arabia.

**Methods:**

Cytogenetic analysis of 171 consent patients with RPL was performed by the standard method of 72-h lymphocyte culture and GTG banding. Allelic polymorphisms of three thrombophilic genes (Factor V Leiden, Prothrombin A20210G, MTHFR C677T) were performed using PCR-RFLP (restriction fragment length polymorphism) and gel electrophoresis.

**Results:**

Data analysis revealed that 7.6 % of patients were carrier of numerical or structural chromosomal abnormalities. A high rate of translocations (46 %) was associated to increased incidence of RPL. A significant correlation between consanguineous RPL patients and chromosomal abnormalities (*P* < 0.05) was found. Both Factor V Leiden and Prothrombin A20210G allelic polymorphisms were significantly associated with a higher prevalence of RPL.

**Conclusions:**

This study demonstrated a strong association between RPL and the prevalence of chromosomal abnormalities and inherited thrombophilia. Given the high rate of consanguineous marriages in the Saudi population, these results underline the importance of systematic cytogenetic investigation and genetic counseling preferably at the premarital stage or at least during early pregnancy phase through preimplantation genetic diagnosis (PGD).

## Background

Recurrent miscarriages (RM) are clinically detectable pregnancies that fail to progress. They are common pregnancy complications that affects 15–20 % of couples [1]. It is a common obstetric health concern that affects around 5 % of women at the reproductive age [2, 3]. Regarding the RM etiology, it is due to several causes including chromosomal, genetic, anatomic, immune and infective factors [1]. Although it is still a controversy whether RM is considered after 2 pregnancy losses versus 3 or more, most of clinicians recommended initiating evaluations from the onset of the second miscarriage since there was no significant difference in RM susceptibility between several patients with 2 versus 3 and more pregnancy losses [4].

Despite worthy studies in Obstetrics/Gynecology clinics and IVF (in vitro fertilization) centers worldwide of this sporadic complication of early pregnancy, RM etiology remains poorly understood [5, 6]. Therefore, effective diagnosis and prevention/treatment approaches are still lacking [7, 8]. Several causes underlying this failure to deliver a normal and viable embryo were reported including aged mothers, uterine abnormalities, placental abruption, incompetent cervix, parents' chromosomal and genetic background, immune disorders and/or endocrine imbalances. Among these causes, three in particular were considered as the major factors of RM including: (i) structural and numerical chromosomal abnormalities, (ii) inflammatory and autoimmune disorders, and (iii) allelic polymorphisms of some pro-thrombophilic genes [6, 9–12]. In fact, positive correlations were reported between chromosomal abnormalities in the embryos and higher RM [13]. This fetal genomic incompatibility to life was associated to chromosomal aneuploidies and mosaicism within the embryo/abortus [14–17]. Moreover, couples who carry chromosomal abnormalities were found to be at risk for repeated miscarriages and therefore have lower chances to deliver a viable offspring [7, 18–21]. Spontaneous miscarriages caused by the chromosomal abnormalities may arise from one of the parents producing defective gametes that will lead to fetal abnormalities and mental disorders. In fact, 3–6 % of RM were due to chromosomal abnormalities of one of the two partners [22–24]. Trisomies in chromosomes 13–16, 21 and 22 were the most common chromosomal aneuploidies followed by monosomy X (45, X). Thus, parental karyotyping is a recommended procedure to assess the cause of recurrent pregnancy losses [23, 25–27].

Pro-thrombophilic factors have also been suggested as one of the major causes of RM. In fact, some genetic polymorphisms of prothrombin (FII G 20210A), Factor V (Factor V Leiden, FVL) and methylene tetrahydrofolate reductase (MTHFR, C677T gene variant) genes were strongly associated with recurrent miscarriages [3, 28, 29]. These factors of inherited thrombophilia disturb normal placental vascularization and formation leading to fetal growth restrictions, pregnancy failure, placental abruption and therefore miscarriages or stillbirth [30].

In addition to the RM incidence worldwide, Saudi Arabia is well known by a high level of consanguineous marriages driven by ethnic or tribal considerations [31]. These consanguineous marriages significantly increase the incidence of inherited recessive disorders and affect some reproductive and developmental health parameters such as infertility rates, recurrent miscarriages, and congenital disorders (e.g. thrombophilia) [32–35]. The objective of this study is to assess possible correlations between chromosomal abnormalities and couples with history of RM. Additional investigations were also carried out to assess the presence of some thrombophilia genetic risk factors including factor V Leiden, Prothrombin A20210G, MTHFR C677T mutations that may further explain the RM outcomes.

## Methods

### Study approach

A cohort of patients with a history of two or more miscarriages up to 20 weeks was conducted at King Abdulaziz University Hospital in the Western region of Saudi Arabia between 2008 and 2013. This study included couples with repeated pregnancy losses who had presented themselves during this period for further investigations. Pregnancy was confirmed by a positive human chorionic gonadotropin (HCG) test using serum or urine in combination with ultrasounds.

### Patients

Following King Abdulaziz University board approval and informed consent of the patients, a cohort of 171 RM patients (73 couples in addition to 25 women only because their husbands were not available for cytogenetic analysis) were selected for peripheral blood collection and a detailed counseling questionnaire covering their personal details, family history and any laboratory results or past investigations that had been conducted. Only patients who experienced two or more miscarriages up to 20 weeks of gestational age were included in this study. Patients’ anonymity and data confidentiality were preserved.

### Cytogenetic analysis

The peripheral blood of each patient (5–10 ml) was subjected to standard 72-h lymphocyte culture to produce Metaphases for cytogenetic analysis (karyotyping) using standard harvesting protocols. GTG banding (G banding) was performed by a pretreatment of chromosomes with trypsin followed by Giemsa staining. Chromosomes’ analysis was done using Cytovision software, a semi-automatic Applied Imaging Karyotyper, and karyotypes were designed according to International System for Human Cytogenetic Nomenclature [36]. Karyotype analysis was performed using at least 20 cells for each patient. In case of suspected mosaicism, this number was expanded to 100 metaphases.

### Genetic risk factors causing thrombophilia

Since associations between thromphobilia and RM were previously reported [37, 38], we proposed in this study to further explore the thrombophilic genetic polymorphisms in our patients’ cohort. The main genes investigated using PCR- RFLP (restriction fragment length polymorphism) were Factor V Leiden (FVL), Prothrombin A20210G and MTHFR C677T. Briefly and following DNA extraction (QIAamp DNA Blood Maxi kit, Qiagen), a Taq-polymerase based PCR using specific primers for each gene was performed (Table 1). The PCR product for each gene is then fragmented using a specific restriction enzyme in order to target potential Single Nucleotide Polymorphisms (SNPs) and separated according to their base pairs’ size by gel electrophoresis as summarized in Table 1.

**Table 1:** Specific primers sequences, restriction enzymes and restriction digestion products’ sizes for FVL, Prothrombin A20210G and MTHFR C677T. The PCR-RFLP products sizes are given according to the genotype polymorphisms

Gene	Length (bp)	Primer’s sequence	Restriction enzyme	Restriction digestion product size (bp)			References
				Normal	Heterozygous	Homozygous	
FVL	143	Forward: CATGAGAGACATCGCCTCTG Reverse: GACCTAACATGTTCTAGCCAGAAG	MnII	25 37 81	25 37 81 118	25 118	[77–80]
Prothrombin	345	Forward: TCTAGAAACAGTTGCCTGGC Reverse: ATAGCACTGGGAGCATTGAAGC	HindIII	345	23 322 345	23 322	
MTHFR	198	Forward: TGAAGGAGAAGGTGTCTGCGGGA Reverse: AGGACGGTGCGGTGAGAGTG	HinfI	198	23 175 198	23 175	

### Statistical analysis

Association between the patients’ clinical features and the cytogenetic analysis in the cohort of patients were assessed using χ2 analysis and Fisher’s exact test. The statistical analysis was carried out using MATLAB R2012a (Version 7.14; The MathWorks, Natick, MA, USA).

## Results

### Patients’ cohort and RM

This study reported the chromosomal analysis of the 171 RM patients (73 couples + 25 women). There were 492 documented pregnancies in this cohort (mean pregnancy per couple = 5.02, SD = 2.79). The age of the subjects ranged from 18 to 48 (mean 32.17, SD = 6.39) (Table 2) and number of miscarriages were between 2 to 14 (mean miscarriage per couple = 4.18; SD = 2.578). Out of 98 women, 32 were able to achieve successful pregnancies which led to viable baby, whereas the remaining 67.35 % were unable to achieve any successful full term pregnancy despite several attempts. Overall, most of clinical pregnancies (79.59 %) were terminated in their first trimester while only 12.24 % and 8.16 % ended up respectively at the second and third trimesters.

**Table 2:** Summary of RM patients’ cohort age range, gender and chromosomal abnormalities incidence

	Total RM patients	Women	Men	Chromosomal abnormalities
No. of cases	171	98	73	13 (7.6 %)
Age range (years)	18 – 48	18 -47	24-48	23-34
Mean	32.17	29.98	35.45	30.50
Std Deviation	6.39	5.98	5.57	4.17
Missing data (%)	16 (9.36 %)	5 (2.92 %)	11 (6.43 %)	1 (0.58 %)

### Chromosomal analysis

Following cytogenetic analysis, 13 patients were found to be carrier of chromosomal abnormalities and/or polymorphisms (7.6 %), 10 of them were women (77 %) and 3 (23 %) were men. The prevalence of mosaicism, balanced translocations, duplications, Robertson translocation, triple X syndrome, and allelic polymorphism were 2.34 %, 1.17 %, 1.17 %, 0.58 %, 0.58 % and 1.17 % respectively. Very surprisingly in this study, the majority of young women (≤35 years) (78.6 %) were carriers of chromosomal aberrations and have a high average of pregnancy failures (Table 3).

**Table 3:** Cytogenetic results, number of miscarriages and age of RM patients with numerical and/or structural chromosomal abnormalities

No.	Sex	Age	Gravidity^a^	Parity^b^	Abortus^c^	Karyotype
1	F	30	8	3	5	45,XX,rob(14:21)(q11.1;q11.1)
2	F	23	2	0	2	46,XX,dup(1)(q11q21)
3	F	34	3	0	3	46,XX,dup(1)(q11q21)
4	F	33	9	2	7	46, XX,t(3;7) (p23;p22)
5	F	28	6	2	4	46,XX[97]/47,XX,+mar[3]
6	M	33	^//^	^//^	^//^	46,XY[76]/47,XY,+mar[4]
7	F	34	4	2	2	46,XX[96]/45,XO[4]
8	M	32	^//^	^//^	^//^	46,XY, add(Y)(p11.3)
9	F	34	4	0	4	46,XX[96]/45,X[2]/37-42,XX,-X,t(7;14) (q34;p10) + mer(cp2)
10	F	24	4	0	4	47,XXX
11	M	-	12	0	12	46,XY,t(3;4;13;6)(q25;q32;q31;q22)
12	F	35	12	0	11	46,XX,13 ps + (polymorphism)
13	F	34	8	2	6	46,XX,16qh + (polymorphism)

^a^Gravidity = No. of pregnancies; ^b^Parity = No. of full term pregnancies; ^c^Abortus = No. of terminated pregnancies

Around 30 % of couples in this patients’ cohort had a family history of consanguineous marriages. Interestingly, a significant positive correlation (*P* = 0.046) between consanguineous marriages and chromosomal abnormalities was recorded. However, correlation between the number of miscarriages and consanguinity was not statistically significant (Table 4). A relatively higher number of spontaneous miscarriages in couples with abnormal karyotype (mean 4.78, SD = 3.11) was observed when compared to those with normal karyotype (mean 4.12, SD = 2.57). Out of 52 pregnancies attempts performed by just 9 couples with chromosomal abnormalities, only 9 (17 %) ended up with live birth.

**Table 4:** Clinical features of RM patients with chromosomal abnormalities

	Normal karyotypes	Chromosomal abnormalities	*P* value
Patient gender			
Male	70	3	0.356
Female	90	8	
Miscarriage stage			
Trimester 1	68	10	0.452
Trimester 2 or 3	19	01	
Miscarriage frequency			
≤ 3	49	4	0.339
> 3	40	7	
Type of marriage			
Consanguineous	51	7	0.046*
Non-consanguineous	109	4	
Citizenship			
Saudi	117	6	0.295
Non Saudi	43	5	

*Significant Fisher exact test (α = 0.05)

### Thrombophilia allelic polymorphisms and RM

Results of SNPs analysis of 3 thrombophilic genes showed that the frequencies of FV Leiden, Prothrombin and MTHFR mutations among RM patients compared to general incidence reported in Saudi population. Allelic polymorphisms of mainly for FVL and Prothrombin genes were relatively high supporting thus our hypothesis of considering these as RM genetic factors (Table 5).

**Table 5:** Prevalence of FV Leiden, Prothrombin and MTHFR mutations among RM patients

Gene	Prevalence in RM patients			Prevalence reported in others studies in Saudi Arabia (%)^c^
	Heterozygous (%)	Homozygous (%)	Total (%)	
FVL	14.92	0.58	15.5^a^	1.3
Prothrombin A20210G	6	-	6^a^	0.7
MTHFR C677T	23.75	1.75	25.5^b^	2.5

^a^Significant (*P* < 0.05); ^b^not significant (*P* > 0.05); ^c^[68]

## Discussions

Recurrent miscarriages are clinically detectable pregnancies that fail to progress due to several causes including chromosomal, genetic, anatomic, immune or infective factors [1]. Chromosomal and genetic abnormalities are among the most common factors leading to recurrent miscarriages and pregnancy demise [23, 39, 40]. Amongst these genetic factors, thrombophilia was shown to be a main cause leading to recurrent miscarriages [3, 29]. Moreover, Saudi population is marked by a high rate of consanguineous marriages (about 58 %), the majority of them were between first-degree cousins [33, 34]. In this particular context, the current study was designed to assess two major parameters know to be causative of RM: (i) the chromosomal abnormalities; and (ii) inherited thrombophilia.

### Chromosomal abnormalities analysis

Following cytogenetic analysis of our patients' cohort, 77 % of carriers of numerical or chromosomal abnormalities were women. It means that a high female to male ratio of 4: 1 in couples carriers of chromosomal abnormalities was recorded, which is higher than previously reported ratios (around 2:1) in Saudi Arabia and elsewhere [18, 41, 42]. This higher ratio in women facing recurrent miscarriages could be explained by the heavy involvement of their oocytes in the fertilization process and early embryo development by providing all the molecular machinery of the new embryo start-up and early development [43–45]. Therefore, these molecular or cytogenetic imbalances look to affect the onset and/or the stability of the pregnancy with higher incidence in women compared to men. These findings are in line with previous reports describing associations between the maternal chromosomal status and RM [46, 47].

Among the carriers’ cohort, more than 46 % of patients showed various types of translocations. This is consistent with previous reports where chromosomal aberrations, mainly translocations in the parents or the abortus (embryo), were shown to be strongly associated with higher incidence of miscarriages [15, 46, 48–52].

The presence of chromosomal polymorphisms were associated with abnormalities of the heterochromatin constitutively located in different loci of the chromosomes and might be associated with some diseases as infertility and RM [53–55].

**Fig. 1:**
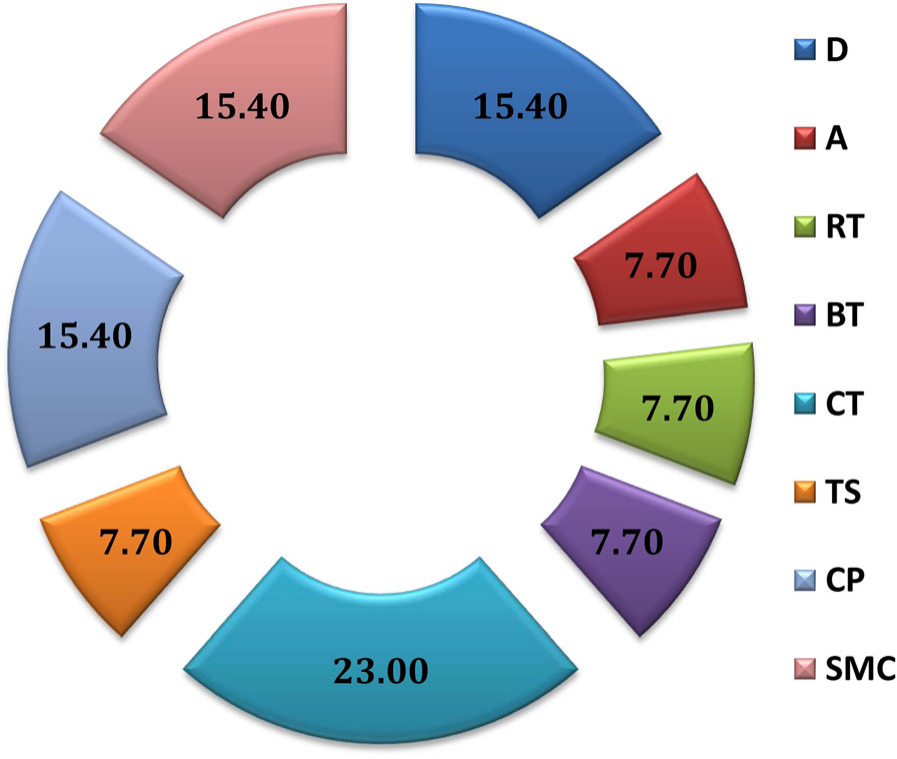
Incidence of numerical and structural chromosomal rearrangements in patients with RM (%). (D): duplications; (A): Additions; (RT): Robertsonian Translocations; (BT): Balanced Translocations; (CT): Complex Translocations; (TS): Turner Syndrome; (CP) Chromosomal Polymorphisms; (SMC): Supernumerary Marker Chromosomes

Turner Syndrome (TS) is another chromosomal disorders reported in our patients’ cohort (Fig. 1) which usually is marked by a total or partial loss of one of the two X chromosomes. In fact, recurrent miscarriages, fetal perinatal death or malformed newborns are known to be frequent in TS patients [56–58]. Our data also confirm this strong association between women with TS and RM although the number of patients carrying this syndrome is not as expected in our cohort, since patients with TS are known to face RM and are not routinely referred for that. Concomitant with our results, other chromosomal disorders, including duplications and deletions have been also reported in RM couples [17, 59].

### Age, consanguinity and RM

The age range of the patients’ group involved in this study was wide (from 18 to 48) (Table 2). One striking finding is that the ages of all of the 13 patients (100 %) identified as carriers of chromosomal abnormalities were below 35 years (Table 3). Despite their relative young age of marriage, these couples were struggling to conceive since they (both or one of them) are carrier of chromosomal abnormalities leading to higher risk of repeated miscarriages and lower chances to deliver a viable and healthy offspring [18, 19]. These patients are also facing an important social pressure to procreate [60] especially in conservative societies as Saudi Arabia. Such particular context may explain the high average (≈5) of pregnancy attempts per couple even at relatively young age. Such social pressure to procreate is very interesting to highlight compared to other societies where the childfree is one of the new reproductive lifestyle trends [60, 61]. However, the high incidence of population inbreeding and consanguineous marriages could explain the high rate of RM at this early age [31, 62]. In fact, around one third of the couples involved in this study were consanguineous (Table 4). Although it is somehow decreasing, this consanguinity driven by an old Arabic tradition is known to rise the frequency of recessive genetic diseases as well as reproductive and developmental disorders such as infertility rates, recurrent miscarriages, and congenital disorders (e.g. thrombophilia) [32–35]. A significant positive correlation between consanguinity and reported chromosomal aberrations and polymorphic variants (*P* = 0.046) documented in this study supports that the RM is mainly due to genomic instability manifested in several chromosomal abnormalities in this group (age ≤ 35) rather than reproductive aging or other known factors. On the other hand, no significant correlation between the number of RM and consanguinity was reported. This result might be explained by the fact that the consanguinity negative effect is observed mainly in the presence of carried chromosomal and/or genetic abnormalities in one or both partners, and only very large cohorts of patients might detect such impact.

However, the aging process seems to be the main cause of RM in couples beyond 35 years since no chromosomal disorders have been detected. In fact, the miscarriage frequency and subsequent reproductive failure were positively correlated to the increase of paternal and/or maternal age [63–65]. Such aging process (beyond 35 and 40 years respectively for women and men) is known to cause genetic and chromosomal disorders during gametogenesis, fertilization and early embryonic development [15, 46, 65]. Such fetal genetic alterations induce a genomic instability and therefore RM.

Out of 52 pregnancies among couples carrying chromosomal abnormalities, only 17 ended up with live birth. This rate is lower than that reported in other studies which showed up to 45 % live birth among couples with structural chromosomal abnormalities. This could be explained by predisposition of the Saudi society to other RM risk factors as thrombophilia [35, 66].

### Thrombophilia genetic polymorphisms and RM

Coagulation anomalies are reported to induce important pregnancy complications. In this context, pro-thrombophilic factors were reported to be involved in RM including FVL, Prothrombin A20210G and MTHFR C677T. These factors are known to disrupt key events associated to placentation, fetal development and pregnancy progression till the delivery [3, 28–30, 67]. In this study, PCR- RFLP was used for molecular analysis of potential SNPs in three (3) thrombophilia-associated genes: FVL, Prothrombin A20210G and MTHFR C677T as detailed in Table 1. The screening for potential SNPs in these 3 genes showed that the frequencies of FVL, Prothrombin and MTHFR mutations (including both homozygous and carriers) were respectively 15.5 %, 6 % and 25.5 (Table 5). These results confirm the atypical and relatively high incidence of thrombophilic gene polymorphisms among Saudi population reported in previous studies [68]. Beside national awareness campaigns, these findings support more preventive measurements to be considered at the premarital and/or before the IVF procedures in ART clinics.

The prevalence of FVL and Prothrombin A20210G mutations reported in patients’ cohort support a strong relationship between these traits and RM. Following analysis of the most important studies about inherited thrombophilia, our data are in line with previous findings where both mutations have been known as common genetic disorders that predispose to early and late RM [3, 29, 69–71]. A recent study in Saudi Arabia also confirmed the involvement of mutations in these two particular genes (FVL and Prothrombin A20210G) in increasing RM incidence [72]. Additionally, these two thrombophilic genetic traits were associated with obstetric complications including miscarriages, placental abruption, intrauterine growth retardation (IUGR) or death [69, 73–75]. These findings explain the significant correlations with RM reported in this study and support the assumption that both factor V Leiden and Prothrombin mutations are major risk factors for RM. Suitable treatment of inherited and acquired thrombophilia will improve the pregnancy outcomes as discussed elsewhere [69]. Therefore, a national medical program for routine screening of these two genes in patients with repeated pregnancy failure in Saudi Arabia is highly recommended.

For MTHFR C677T mutation in RM patients and in agreement with other studies, no significant (*P* > 0.05) association with RM were found [29]. The general practice of folic acid supplementation during pregnancy seems to be the reason of masking the effect of the MTHFR mutation in RM patients as reported elsewhere [76].

Since RM is a challenging obstetric complication with various psychological, societal and economic burdens on both couples and the health care system in general, our study contributed to report an effect of both structural and numerical effects of chromosomal abnormalities on RM, which were amplified by consanguinity mainly for couples under 35 years. Moreover, thrombophilic polymorphisms of FVL and Prothrombin A20210G were significantly associated to higher prevalence of RM.

## Conclusions

This study demonstrated a strong association between RPL and the prevalence of chromosomal abnormalities and inherited thrombophilia and confirms the high incidence of RM in the Western region of Saudi Arabia suggesting thus some main but non-exclusive causes of this disease. Our findings lay also foundation for larger cohort-based studies to further validate and confirm the impact of thalassemia gene polymorphisms and hemoglobinopathies in general, chromosomal aberrations and consanguinity, but also to predict the involvement of other anatomic, endocrine or auto-immune factors.

Our study highlights the importance of including cytogenetic and thrombophilia testing as part of the routine clinical investigation of RM and during Preimplantation Genetic Screening (PGS) in IVF clinics in order to suggest suitable management and/or treatment approaches. Such genetic testing along with standard karyotyping are highly recommended to be included in premarital test especially for consanguineous partners. In the genomic era, further studies focusing on the molecular mechanism of thrombophlic polymorphisms on placental pathologies and pregnancy loss using high-throughput technologies as array Comparative Genomic Hybridization (aCGH) and Next Generation Sequencing (NGS) are highly recommended. We believe that a multidisciplinary and collaborative approach between obstetricians, geneticists, hematologists, scientists and bioethicists combined with effective awareness program will contribute to relieve the burden of RM.

## Abbreviations

FVL: Factor V Leiden
HCG: Human chorionic gonadotropin
IVF: In vitro fertilization
PGD: Preimplantation genetic diagnosis
RFLP: Restriction fragment length polymorphism
RM: Recurrent miscarriages
RPL: Recurrent pregnancy loss
SNPs: Single nucleotide polymorphisms
TS: Turner syndrome
